# Lightweight Single Image Super-Resolution via Efficient Mixture of Transformers and Convolutional Networks

**DOI:** 10.3390/s24165098

**Published:** 2024-08-06

**Authors:** Luyang Xiao, Xiangyu Liao, Chao Ren

**Affiliations:** College of Electronics and Information Engineering, Sichuan University, Chengdu 610065, China; xiaoluyang@stu.scu.edu.cn (L.X.); liaoxiangyu1@stu.scu.edu.cn (X.L.)

**Keywords:** image super-resolution, efficient global interaction, fine-grained local modeling

## Abstract

In this paper, we propose a Local Global Union Network (LGUN), which effectively combines the strengths of Transformers and Convolutional Networks to develop a lightweight and high-performance network suitable for Single Image Super-Resolution (SISR). Specifically, we make use of the advantages of Transformers to provide input-adaptation weighting and global context interaction. We also make use of the advantages of Convolutional Networks to include spatial inductive biases and local connectivity. In the shallow layer, the local spatial information is encoded by Multi-order Local Hierarchical Attention (MLHA). In the deeper layer, we utilize Dynamic Global Sparse Attention (DGSA), which is based on the Multi-stage Token Selection (MTS) strategy to model global context dependencies. Moreover, we also conduct extensive experiments on both natural and satellite datasets, acquired through optical and satellite sensors, respectively, demonstrating that LGUN outperforms existing methods.

## 1. Introduction

Single Image Super-Resolution (SISR) is a prominent research field in computer vision that focuses on enhancing the visual details and overall appearance of low-resolution (LR) images by generating high-resolution (HR) versions. It has diverse applications across domains such as surveillance [1,2,3,4], medical imaging [5,6], satellite imagery [7,8], and monitoring [9,10]. Recent advancements in SISR techniques have leveraged advanced algorithms and deep learning models to effectively recover missing high-frequency details and textures from LR inputs, enabling significant improvements in resolution and visual quality.

Convolutional Networks are widely adopted for various visual tasks, including SISR [11,12]. The inherent properties of convolutional operations, such as the ability to aggregate information from adjacent pixels or regions, e.g., 3 × 3 windows, make them effective at capturing spatially local patterns. These properties, including translation invariance, local connectivity, and the sliding-window strategy, provide valuable inductive biases. However, Convolutional Networks suffer from two main limitations. Firstly, they have a local receptive field, restricting their ability to model global context. Secondly, the interaction between spatial locations is fixed through a static convolutional kernel during inference, limiting their flexibility to adapt to varying input content. Transformers, on the other hand, offer a solution to address these limitations. By introducing self-attention (SA) in Vision Transformers (ViTs), global interactions can be explicitly modeled, and the importance of each token can be dynamically adjusted through attention scores computed between all pairs of tokens during inference. However, the computational complexity of Transformers, which grows quadratically with the token length *N* (or spatial resolution HW), poses challenges for real-world applications on resource-constrained hardware. This leads to the following natural question: How can we effectively combine the strengths of Convolutional Networks and ViTs to develop a lightweight and high-performance network suitable for resource-constrained devices?

**Figure 1:**
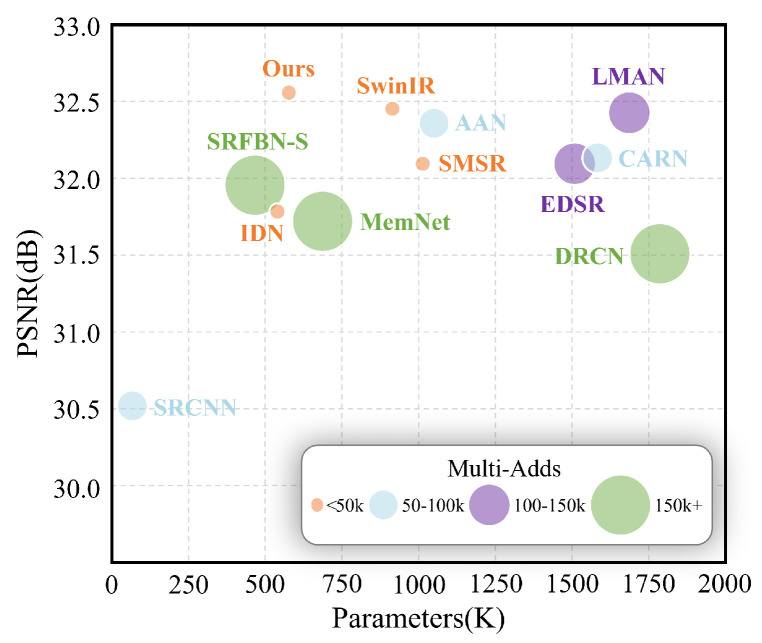
Trade–off between performance and model complexity on Set5 ×4 dataset. Multi-Adds are calculated on 1280×720 HR images.

**Figure 2:**
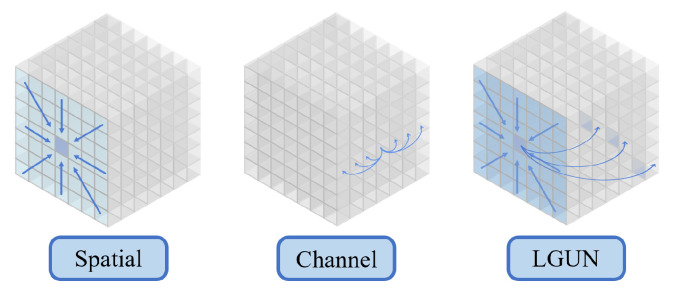
Compared to uni-dimensional information communication, e.g., spatial-only or channel-only, our method can achieve local spatial-wise aggregation and global channel-wise interaction simultaneously, both of which are crucial for SISR tasks.

**Figure 3:**
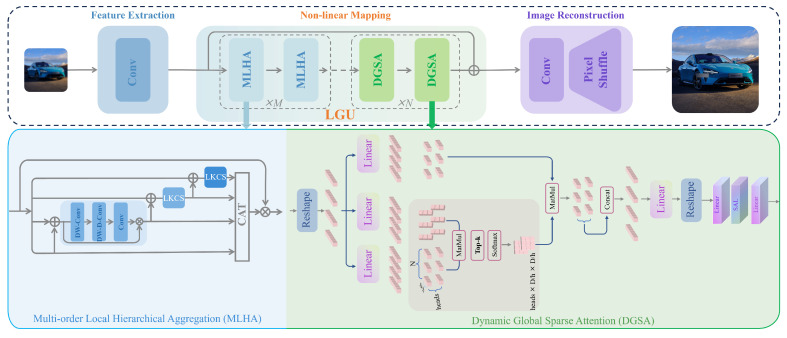
The architecture of our proposed method, LGUN, consists of three main parts: feature extraction, nonlinear mapping, and image reconstruction. The core modules, named LGU, include two stages: MLHA and DGSA. In the shallow layers, MLHA efficiently encodes local spatial information by utilizing subsets of the entire feature, enabling explicit learning of distinct feature patterns through the STF strategy. In the deep layers, DGSA is employed to model long-range non-local dependencies while achieving a global effective receptive field. DGSA operates across the feature dimension and leverages interactions based on the cross-covariance matrix between keys and queries. Moreover, we incorporate the MTS strategy into DGSA, which selects multiple top-*k* similar attention matrices and masks out elements with lower weights. This reduces redundancy in attention maps and suppresses interference from cluttered backgrounds. LGUN exhibits robustness to changes in the input token length and significantly reduces the computational complexity to $O(NC^{2})$, where C≪N.

In this work, we address the aforementioned question by focusing on the design of a lightweight and high-performance network for SISR tasks. The performance of our work is shown in Figure 1 compared with others. Our proposed approach, named LGUN, leverages the advantages of Convolutional Networks, such as spatial inductive biases and local connectivity, as well as Transformers, which offer input-adaptation weighting and global context interaction. Therefore, our core concept is illustrated in Figure 2. Compared to uni-dimensional information communication, e.g., spatial-only communication such as EIMN [13] or channel-only communication such as Restormer [14], our method can achieve local spatial-wise aggregation and global channel-wise interaction simultaneously, both of which are crucial for SISR tasks. As commonly known, in Convolutional Networks, the shallow layers of a network employ convolutional filters with smaller receptive fields, capturing local patterns and features like edges, corners, and textures. These low-level features are extracted in the initial layers, providing local information about the input data. By stacking multiple building blocks, Convolutional Networks gradually enlarge their receptive fields, enabling the capture of large-range spatial context information. Based on this prior knowledge, as shown in Figure 3, we divide the core modules, named Local Global Union (LGU), into two stages: Multi-order Local Hierarchical Aggregation (MLHA) and Dynamic Global Sparse Attention (DGSA). In the shallow layers, we employ MLHA to encode local spatial information efficiently. This approach feeds each sub-branch with only a subset of the entire feature, facilitating the explicit learning of distinct feature patterns through the Split–Transform–Fusion (STF) strategy. In the deep layers, we introduce DGSA to model long-range non-local dependencies while obtaining an effective receptive field of H × W. DGSA operates across the feature dimension, utilizing interactions based on the cross-covariance matrix between keys and queries. Considering the potential negative impact of irrelevant or confusing information in the attention matrix, which other methods [14] fail to consider, we incorporate the Multi-stage Token Selection (MTS) strategy into DGSA, which selects multiple top-*k* similar attention matrices and masks out insignificant elements allocated with lower weights. This reduces redundancy in attention maps and suppresses interference from cluttered backgrounds. The proposed design is robust to changes in the input token length and decreases the computational complexity to $O(NC^{2})$, where C≪N.

Our contributions can be summarized as follows:

(1) We propose LGUN, a hybridization structure designed for resource-constrained devices. It combines the strengths of Convolutional Networks and ViTs, allowing for effective encoding of both local processing and global interaction throughout the network by the proposed LGU.
(2) In the shallow layer, we employ MLHA to focus on encoding local spatial information. By using the STF strategy, MLHA promotes the learning of different patterns while also saving computational resources. In the deep layer, we utilize DGSA based on the MTS strategy to model global context dependencies. This enhances the network’s ability to model complex image patterns with high adaptability and representational power.
(3) Experimental results on popular benchmark datasets demonstrate the superiority of our method compared to other recently advanced Transformer-based approaches. Our method outperforms in both quantitative and qualitative evaluations, providing evidence for the effectiveness of the MLHA-with-STF strategy and the DGSA-with-MTS strategy.

## 2. Related Work

### 2.1. Convolutional Networks

**Classical SISR.** Since the introduction of SRCNN [15], Convolutional Networks have emerged as superior solutions for SISR tasks [16]. Over the past decade, numerous novel ideas have been proposed or introduced in this field. These include residual learning [11], densely connected networks [17], neural architecture search (NAS) [18], knowledge distillation [19], channel attention [20], spatial attention [21], non-local attention [22], SA [23], etc. The general trend towards achieving higher performance in SISR is to design deeper and more complex networks. However, these methods often come at the cost of increased computational requirements, making it challenging to deploy them on resource-constrained mobile devices for practical applications.

**Efficient SISR.** To make Convolutional Networks suitable for computationally limited platforms such as mobile devices, methods such as pruning, NAS, knowledge distillation, reparameterization, and efficient design of convolutional layers have been proposed. Pruning technology involves removing insignificant connections or neurons from a network to reduce its size and complexity, thereby improving generalization ability and computational speed. NAS technology [24], on the other hand, automates the search for the optimal neural structure by exploring various combinations of structures across different platforms with varying computational capabilities. Knowledge distillation technology [19], a method for training smaller models, transfers knowledge from larger, more complex models to enhance performance while reducing computational requirements. Structural reparameterization [25] technology utilizes a multi-branch architecture during training and switches to a plain network during testing to achieve faster inference speed. Efficient convolutional layers, such as depth-wise convolution [26] and convolutional factorization [27], reduce computational resources while maintaining high performance. These design concepts have significantly contributed to the advancement of SISR. However, many existing methods either focus on local spatial information and lack global context understanding, or have high computational complexity that limits their applicability to edge devices. In this work, we propose a hybrid structure called LGUN that combines the strengths of Convolutional Networks (e.g., spatial inductive biases and local connectivity) and Transformers (e.g., input-adaptive weighting and global context processing). Notably, our approach achieves a superior trade-off between complexity and performance (Parameters/Multi-Adds @ PSNR/SSIM: 675K/141G @ 38.24/0.9618).

### 2.2. Transformers

**Pioneer work.** Recently, Transformers have attracted significant interest in the computer vision community, thanks to their success in natural language processing (NLP) field. Several studies have explored the benefits of using a Transformer in vision tasks, e.g., FAT [28] and RISTRA [29]. The seminal work, Vision Transformer (ViT) [30], applies a standard Transformer architecture directly to 2D images for visual recognition and demonstrated promising results. The Image Processing Transformer (IPT) [23] leverages the power of the Transformer to achieve superior performance on various image restoration tasks, such as SR, denoising, and deraining. However, the quadratic computational cost make it difficult to apply the SA mechanism to the SISR task.

**Efficient Transformers.** Numerous efforts have been made to reduce complexity and maintain performance in order to make Transformers more suitable for vision tasks. For instance, Swin Transformer [31] and SwinIR [32] limit the SA calculation to non-overlapping local windows instead of the global scope and introduce a shift operation for cross-window interaction. This approach significantly reduces computational complexity on HR feature maps while capturing local context. Similarly, shuffle Transformer [33] and HaloNet [34] utilize spatial shuffle and halo operations, respectively, instead of shifted window partitioning. MobileViT [35] employs element-wise operations as replacements for computationally and memory-intensive operations, such as batch-wise matrix multiplication and softmax, to compute context scores. Linformer [36] substitutes self-attention with low-rank approximation operations. Axial self-attention [37] achieves longer-range dependencies in the horizontal and vertical directions by performing SA within each single row or column of the feature map. CSWin [38] proposes a cross-shaped window SA region that includes multiple rows and columns, while Pale Transformer [39] performs SA within a pale-shaped region composed of the same number of interlaced rows and columns of the feature map. Although these methods achieve a trade-off in performance across various vision tasks, the dependencies in the SA layer are limited to local regions to reduce computational complexity, resulting in insufficient context modeling. This limitation restricts the modeling capacity of the entire network. In this study, we propose DGSA, which models long-range non-local dependencies while achieving an effective receptive field of H × W that operates across the feature dimension. The interactions are based on the cross-covariance matrix between keys and queries. Importantly, the computational complexity is only linear, $O(NC^{2})$, rather than quadratic, $O(N^{2}C)$, where *C* is much smaller than *N*.

**Sparse Transformers.** In addition, the utilization of global-based attention involves computing attention matrices that consider all image patches (tokens), prompting the question of whether it is necessary for all elements in the sequence to be attended. The answer to this query is: *NO!* The inherent dense calculation pattern of the SA mechanism amplifies the weights of relatively lower similarities, rendering the feature interaction and aggregation process susceptible to implicit noise. Consequently, redundant or irrelevant representations continue to influence the modeling of global feature dependencies. Numerous studies have demonstrated that the adoption of sparse attention matrices can enhance model performance while reducing memory usage and computational requirements. For instance, Sparse Transformer [40] employs a factorized operation to mitigate complexity and suggests reducing the spatial dimensions of attention’s key and value matrices. Explicit Sparse Transformer [41] improves attention concentration on the global context by explicitly selecting the most relevant segments in natural language processing (NLP) tasks. EfficientViT [42] further addresses redundancy in attention maps by explicitly decomposing the computation of each head and feeding them with diverse features. In this study, instead of computing the attention matrix for all query–key pairs as in the conventional SA mechanism, we adopt a selective approach in the proposed DGSA. Specifically, we choose the top-*k* most similar keys and values for each query. However, the use of predefined *k* values can be seen as a form of hard coding, potentially impeding the relational learning between pairwise pixels. To mitigate this issue, we generate multiple attention matrices with different degrees of sparsity by employing multiple *k* values. These matrices are then weighted by adaptively learned coefficients for fusion. Our approach can give higher attention to high-contributing regions while giving stronger suppression to low-contributing regions.

### 2.3. Combination of Transformers and Convolutional Networks

Several works have incorporated classical design principles of Convolutional Networks into Transformers. These include (1) preserving locality property [43,44,45,46,47,48] and (2) adopting specific network architectures such as U-Net [14,49,50,51], hierarchical pyramid-like structures [52,53,54], and two-stream architectures [55]. On the other hand, MobileViT [35] and MobileFormer [56] successfully combine MobileNet [57] and ViT [30] to achieve competitive results on mobile devices. HAT [58] introduces a hybridized network with parallel branches for channel attention and multi-head self-attention (MHSA) to reconstruct individual pixels or small regions. ACT [59] utilizes both Transformer and convolution branches and implements a fuse–split strategy to efficiently aggregate local–global information at each stage. In this work, we propose a novel hybridization structure, named LGUN, which leverages the advantages of Convolutional Networks, such as spatial inductive biases and local connectivity, and combines them with Transformers’ input-adaptive weighting and global context processing. By encoding shallow, fine-grained local information and effectively interacting with deep global contextual information, our approach achieves a higher complexity–performance trade-off (Parameters/Multi-Adds @ PSNR/SSIM: 542K/113G @ xxx).

## 3. Methods

### 3.1. Overall Architecture

The proposed network architecture consists of three primary components: (1) feature extraction FE(·), (2) nonlinear mapping NLM(·), and (3) reconstruction REC(·). The input and output of the model are denoted as $I_{\mathrm{LR}}\in R^{H\times W\times 3}$ and $I_{\mathrm{SR}}\in R^{H\times W\times 3}$, respectively. In the initial stage, $I_{\mathrm{LR}}$ undergoes an overlapped image patch embedding process, where a 3 × 3 convolution layer is applied at the beginning of the network. This results in $F_{\mathrm{embed}}\in R^{H\times W\times C}$ feature maps. Subsequently, $F_{\mathrm{embed}}$ passes through *N* stacked blocks to facilitate the learning of local and global relationships. The final reconstructed result is obtained as follows: $I_{\mathrm{SR}}=\mathrm{REC}\left(\mathrm{NLM}\left(F_{\mathrm{embed}}\right)+F_{\mathrm{embed}}\right)$.

### 3.2. LGU

The core modules of LGU, as depicted in Figure 3, include Multi-order Local Hierarchical Aggregation (MLHA) and Dynamic Global Sparse Attention (DGSA). The MLHA module efficiently encodes local spatial information by feeding each sub-branch with a subset of the entire feature, facilitating the explicit learning of distinct feature patterns. On the other hand, the DGSA module aims to model long-range non-local dependencies by leveraging interactions across feature dimensions, resulting in an effective global receptive field. This design ensures robustness to changes in the input token length while reducing computational complexity to $O(NC^{2})$, where C≪N. More specific details are provided below:

(1) $$\begin{array}{cc} \; & Shallow\;Layer\left\{\begin{array}{cc} X^{\prime } & =X+MLHA(Norm(X))\\ X^{\prime \prime } & =X^{\prime }+FFN\left(Norm\left(X^{\prime }\right)\right) \end{array}\right. \end{array}$$

(2) $$\begin{array}{cc} \; & Deep\;Layer\left\{\begin{array}{cc} Z^{\prime } & =Z+DGSA(Norm(Z))\\ Z^{\prime \prime } & =Z^{\prime }+FFN\left(Norm\left(Z^{\prime }\right)\right) \end{array}\right. \end{array}$$

### 3.3. Multi-Order Local Hierarchical Aggregation (MLHA)

In the shallow layer of our method, we employ MLHA to focus on encoding local spatial information. By using the Split–Transform–Fusion (STF) strategy, MLHA promotes the learning of different patterns while also saving computational resources.

Given the input feature $X\in R^{H\times W\times C}$, it passes through three consecutive units: Linear–MLHA–Linear. The specific details of MLHA are as follows:

Firstly, split. The input feature $F_{\mathrm{in}}\in R^{H\times W\times C}$ is divided into *m* subparts denoted by $x_{i}$. Each subpart has the same spatial size of H×W and a channel number of $\frac{1}{s}C$, where $i\in \left\{1,2,...,m\right\}$.

Secondly, transform. Each subpart feature $x_{i}$ is individually processed by a large kernel convolutional sequence (LKCS) denoted as $LKCS_{i}\left(\cdot \right)$, which performs self-adaptive recalibration of the subpart features. Each $LKCS_{i}\left(\cdot \right)$ has a similar structure: ${\mathrm{DW}\text{-}\mathrm{Conv}}_{k_{1}\times k_{1}},{\mathrm{DW}\text{-}D\text{-}\mathrm{Conv}}_{k_{2}\times k_{2}}$, and ${\mathrm{Conv}}_{k_{3}\times k_{3}}$.

Finally, fusion. The MLHA integrates multiple re-weighting $LKCS_{i}\left(\cdot \right)$ processes, enabling the modeling of spatial pixel relationships and the interaction of multi-order context information for input content self-adaptation. Specifically, each subpart feature $x_{i}$(i>1) is added to the output of $LKCS_{i-1}\left(\cdot \right)$ and then passed to the next branch $LKCS_{i}\left(\cdot \right)$ for further processing. The output feature $y_{i}$ of $LKCS_{i}\left(\cdot \right)$ corresponds to the input $x_{i}$ and is passed to the concatenation layer. The concatenation layer aggregates large-range spatial relationships and multi-order context information, treating them as weight matrices for self-adaptive modulation of the input feature $F_{\mathrm{in}}$. By effectively mining the underlying relevance of $F_{\mathrm{in}}$, positions with high scores receive adequate attention while insignificant positions are suppressed. This flexible and effective modulation of the feature representation promotes the modeling of complex image patterns with high adaptability and representational power. The process can be expressed as follows:

(3) $$F_{\mathrm{MLHA}}=F_{\mathrm{in}}⊙Concat\left(y_{1},...,y_{s}\right)$$

(4) $$y_{i}=\left\{\begin{array}{cc} x_{i}, & i=1;\\ LKCS_{i}\left(x_{i}+y_{i-1}\right), & 1<i\leq s \end{array}\right.$$

### 3.4. Dynamic Global Sparse Attention (DGSA)

The token-based SA mechanism calculates the weight matrix along the token dimension. However, the quadratic increase in computational complexity as the sequence length N grows makes it unsuitable for long sequences and high-resolution images. To address this, compromise solutions have been proposed with two approaches: (1) replacing global SA with local SA, which restricts the SA calculation to local windows, and (2) reducing the sequence length of the key and the value through pooling or stride convolution. However, the former method can only capture dependencies within a limited local range, thus constraining the modeling capacity of the entire network to a local region. The latter method, on the other hand, may result in excessive downsampling, leading to information loss or the confusion of relationships, which contradicts the purpose of SISR. In this work, we present an efficient solution that enables global interactions in SA with linear complexity. Instead of considering global interactions between all tokens, we propose the use of Dynamic Global Sparse Attention (DGSA), which operates across feature channels rather than tokens. In DGSA, the interactions are based on the cross-covariance matrix computed over the key and query projections of the token features. The specific details are as follows:

Consider an input token sequence, $X\in R^{N\times D}$, where *N* and *D* denote the length and dimension of the input sequence, respectively. DGSA first generates the query Q, key K, and value V using linear project layers from X,

(5) $$Q=XW_{q},K=XW_{k},V=XW_{v}$$

where $W_{q}$, $W_{k}$, and $W_{v}\in R^{D\times D_{h}}$ are learnable weight matrices and $D_{h}$ is the number of project dimensions. Next, the output of DGSA is computed as a weighted sum over *N* value vectors,

(6) $$A\left(Q,K,V\right)=V\cdot Softmax\left(\frac{K^{⊤}\cdot Q}{\sqrt{d_{h}}}\right)$$

Importantly, DGSA has a linear complexity of O(N) rather than $O(N^{2})$ in vanilla SA.

**Figure 4:**
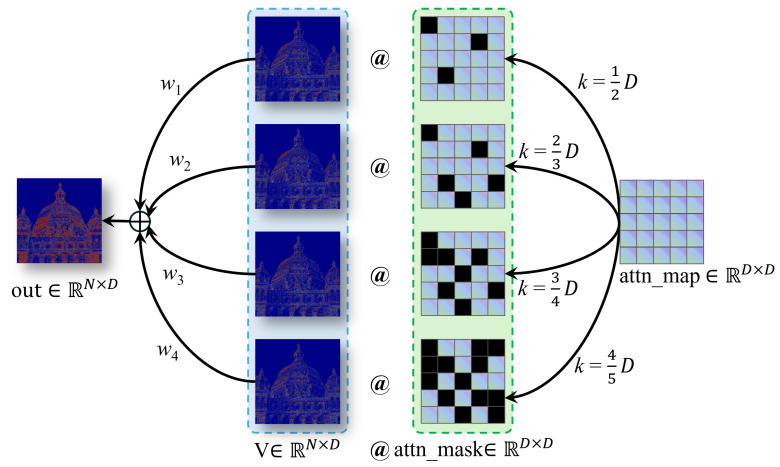
Multiple attention matrices. Take a head as an example ($D=D_{h}$), where $w_{1}$, $w_{2}$, $w_{3}$, and $w_{4}$ represent the assigned weight, which is obtained by dynamic adaptation learning of the network. We set Multi-stage Token Selection thresholds $k_{1}$, $k_{2}$, $k_{3}$, and $k_{4}$ to $\frac{1}{2}$, $\frac{2}{3}$, $\frac{3}{4}$, and $\frac{4}{5}$, respectively.

As mentioned in the Introduction, to address the potential negative impact of irrelevant or confusing information in the SISR task, we introduce a Multi-stage Token Selection (MTS) strategy. As shown in Figure 4, this strategy involves selecting the top-*k* similar tokens from the keys for each query in order to compute the attention weight matrix. To achieve this, we employ multiple different *k* values parallelly, resulting in multiple attention matrices with varying degrees of sparsity. The final output is obtained by combining these matrices through a weighted sum. The DGSA with MTS can be expressed as follows:

(7) $$DGSA\left(Q,K,V\right)=\sum_{n=1}^{3}w_{n}*DGSA_{k_{n}}\left(Q,K,V\right)$$

(8) $$DGSA_{k_{n}}\left(Q,K,V\right)=V\cdot Softmax\left(T_{k_{n}}\left(\frac{K^{⊤}\cdot Q}{\sqrt{d_{h}}}\right)\right)$$

where $w_{1}$, $w_{2}$, and $w_{3}$ represent the assigned weight, which is obtained through dynamic adaptation learning by the network, with an initial value of 0.1, and $T_{k_{n}}\left(\cdot \right)$ is the dynamic learnable row-wise top-*k* selection operator:

(9) $${\left[T_{k}\left(A\right)\right]}_{ij}=\left\{\begin{array}{cc} A_{ij} & \left.A_{ij}\in \;\mathrm{top}-k(\mathrm{row}\;j\right)\\ -inf & \;\mathrm{otherwise}\; \end{array}\right.$$

We set Multi-stage Token Selection thresholds $k_{1}$, $k_{2}$, and $k_{3}$ to $\frac{1}{2}$, $\frac{2}{3}$, and $\frac{3}{4}$, respectively.

In conclusion, DGSA offers two significant advantages. Firstly, it enables the modeling of global correlations by selecting the most similar tokens from the entire attention matrix while effectively filtering out irrelevant ones. Secondly, by employing a weighted sum of multiple attention matrices with varying degrees of sparsity, the model can adequately capture the underlying relevance between all pairs of positions. This approach assigns higher weights to positions of greater importance while suppressing insignificant positions. Consequently, it facilitates the identification of crucial features and their effective utilization in subsequent processing steps. Through this mechanism, our method adaptively selects high-contributing scores from input elements, promoting the modeling of complex image patterns with enhanced adaptability and representational power.

### 3.5. Feed-Forward Network (FFN)

The original Feed-Forward Network (FFN) has limitations in modeling local patterns and spatial relationships, which are crucial for SISR. The inverted residual block (IRB) incorporates a depth-wise convolution between two linear transform layers. This design enables the aggregation of local information among neighboring pixels within each channel. Building upon this idea, we adopted the IRB’s design paradigm, and the point-wise convolutional layers in the vanilla FFN were replaced with a combination of depth-wise convolutions and excitation-and-squeeze modules. This modification captures local patterns and structures effectively. Further details are provided below.

(10) FFN(X)=Linear(σ(SAL(Linear(X))))

where σ indicates the nonlinear activation function GELU. SAL indicates the spatial awareness layer.

### 3.6. Discussion

As mentioned earlier, our method combines the strengths of Convolutional Networks, such as spatial inductive biases and local connectivity, with Transformers, which provide input-adaptive weighting and global context processing. This integration allows us to achieve a favorable balance between complexity and performance. The advantages of our approach can be summarized as follows:

(1) Fine-grained local modeling. The MLHA incorporates a re-weighting process into both the sub-branch and entire features. By utilizing the extracted convolutional features as weight matrices, we can self-adaptively re-calibrate the input representations, effectively capturing spatial relationships and enabling multi-order feature interactions. This approach ensures that important positions receive appropriate focus while suppressing insignificant positions. It is worth noting that each sub-branch feature $x_{i}$ can receive features from all subparts $x_{i}$, j≤i, and passes through large kernel convolutional sequences, resulting in a larger receptive field.

(2) Efficient global interaction. The DGSA is capable of modeling long-range non-local dependencies while obtaining an effective global receptive field. The interactions in DGSA operate across feature dimensions and are based on the cross-covariance matrix between keys and queries. To avoid interference with subsequent super-resolution tasks, our MTS strategy selects multiple top-*k* similarity scores between queries and keys for attention matrix calculation. This strategy masks out insignificant elements with lower weights, reducing redundancy in attention maps and suppressing clutter background interference, thereby facilitating better feature aggregation.

(3) Linear complexity. Our method remains robust to changes in the input token length while achieving linear computational complexity of $O(NC^{2})$, where C≪N. This enables flexible and effective modeling of feature representation, promoting the capture of complex image patterns with high representational power.

## 4. Experiments

### 4.1. Implementation Details

Our proposed method comprises 16 fundamental building blocks, with each block having 64 channels. Minor channel adjustments are made only in the image reconstruction part for the ×2, ×3, and ×4 scales. To evaluate the effectiveness of our proposed method, we tested it on five common benchmark datasets: Set5 [60], Set14 [61], BSD100 [62], Urban100 [63], and Manga109 [64]. We measured the average peak-signal-to-noise ratio (PSNR) and the structural similarity (SSIM) on the luminance (Y) channel of YCbCr space. Our method was implemented using Pytorch 1.12.0 and trained on a single NVIDIA RTX 3090 GPU. More hyper-parameters of the training process are shown in Table 1.

### 4.2. Comparison with State-of-the-Art (SOTA) Methods

To validate the effectiveness of our method, we present the reconstruction results obtained by various SR models on both natural and satellite remote sensing images. These images were captured using common optical sensors (e.g., CMOS) as well as satellite sensors (e.g., millimeter-wave sensors). First, we verify the effectiveness of our proposed method on natural images. In Section 4.2.3, we verify the effectiveness of the method on satellite remote sensing images.

#### 4.2.1. Quantitative and Qualitative Results

In Table 2, we compare the proposed method with recent SOTA efficient SISR approaches for upscale factors of ×2, ×3, and ×4 on five benchmark datasets. For instance, we used SRCNN [15], VDSR [11], DRCN [65], LapSRN [66], MemNet [67], SRFBN-S [68], IDN [69], CARN [70], EDSR [12], FALSR-A [18], SMSR [71], A2N [72], LMAN [26], DRSDN [24], SwinIR [32], and NGswin [73]. Notably, SwinIR [32] and NGswin [73] are recently advanced Transformer-based methods. Specifically, in Set5, the average PSNR value at ×2 scale is improved by 0.63 and the average SSIM value of ×2 scale is improved by 0.0036 on average over other methods; the average PSNR value at ×4 scale is improved by 0.89 and the average SSIM value at ×4 scale is improved by 0.0144 on average over other methods. In Set14, the average PSNR value at ×2 scale is improved by 0.64 and the average SSIM value at ×2 scale is improved by 0.0079; the average PSNR value at ×4 scale is improved by 0.64 and the average SSIM value at ×4 scale is improved by 0.0165 on average over other methods. Obviously, with a lower complexity, **our method (Parameters/Multi-Adds @ PSNR/SSIM: 542K/113G @ 38.24/0.9618)** obtains better PSNR/SSIM results compared to recently improved Transformer-based and Convolutional Network-based methods, such as **SwinIR (878K/243.7G @ 38.14dB/0.9611)** and **NGswin (998K/140.4G @ 38.05dB/0.9610)**.

**Figure 5:**
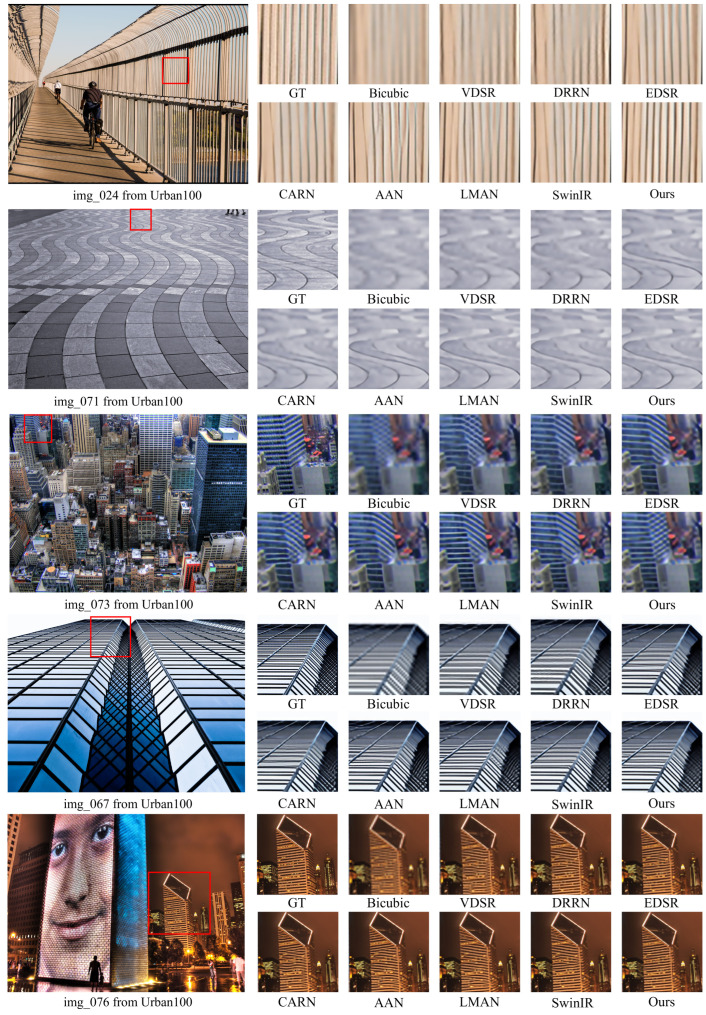
Qualitative comparison of state-of-the-art methods on Urban100 [63]. Our method achieves better performance with fewer artifacts and less blur.

In Figure 5, we present the qualitative comparison results for different methods at upscale factors of ×4. For the images “img 024”, “img 067”, “img 071”, “img 073” and “img 076” in the Urban100 dataset, our method demonstrates superior reconstruction of lattice and text patterns with minimal blurriness and artifacts compared to other methods. This observation confirms the usefulness and effectiveness of our approach. Taking the image “img 024” as an example, our method accurately generates stripes with the correct direction and minimal blurring, while the other methods produce incorrect stripes and a noticeable blur over a wide range.

#### 4.2.2. Visualization Analysis

**Figure 6:**
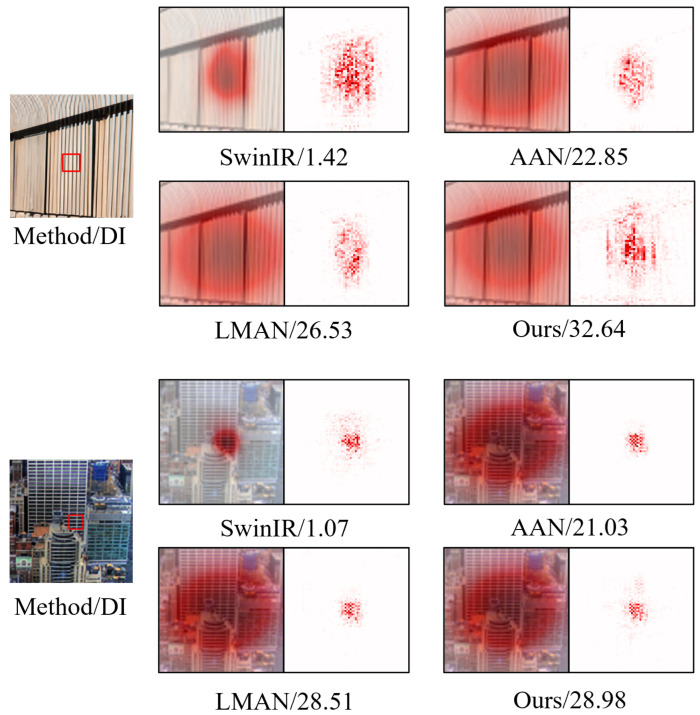
Results of local attribution maps. A more widely distributed red area and higher DI represent a larger range of pixel utilization.

**LAM Results.** In Figure 6, we analyze the local attribution map (LAM [76]) results for SwinIR [32], AAN [72], LMAN [26], and our method to investigate the utilization range of pixels in the input image during the reconstruction of the selected area. We employ the diffusion index (DI) as an evaluation metric to assess the model’s ability to extract features and utilize relevant information. As illustrated in Figure 6, our method exhibits the utilization of a larger range of pixel information in reconstructing the area outlined by a red box. This observation demonstrates that our approach achieves a larger receptive field through an efficient local and global interaction.

**Figure 7:**
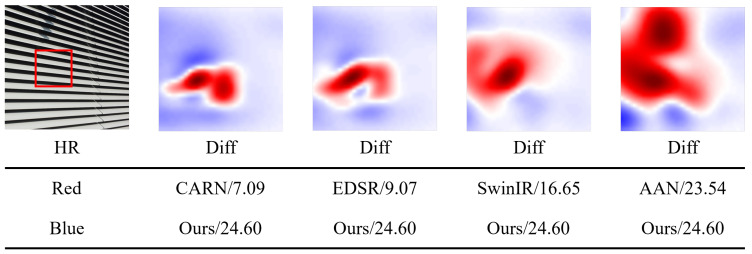
The heat maps exhibit the area of interest for different SR networks. The red regions are noticed by CARN [70], EDSR [12], SwinIR [32] and AAN [72], while the blue areas represent the additional LAM interest areas of the proposed LGUN. (LGUN has a higher diffusion index).

To facilitate intuitive comparisons, we present a heat map, as shown in Figure 7, illustrating the differences in interest areas between the SR networks (referred to as “Diff”). An observation can be made that the proposed LGUN exhibits a more extensive diffusion region compared to CARN [70], EDSR [12], SwinIR [32], and AAN [72]. This observation indicates that our designs enable the exploitation of a greater amount of intra-frame information while maintaining limited network complexity. This is primarily attributed to the MLHA and DGSA employed in LGUN, which facilitate the learning of diverse information ranges and the selective retention of spatial textures deemed useful.

#### 4.2.3. Remote Sensing Image Super-Resolution

Satellite sensors play a vital role in remote sensing by capturing images and data of the Earth’s surface from space. These sensors are mounted on Earth-orbiting satellites and are specifically designed to gather information across multiple wavelengths of the electromagnetic spectrum. Remote sensing images obtained from satellite sensors offer valuable insights for a wide range of applications, including environmental monitoring, land use classification, disaster management, and climate studies.

**Figure 8:**
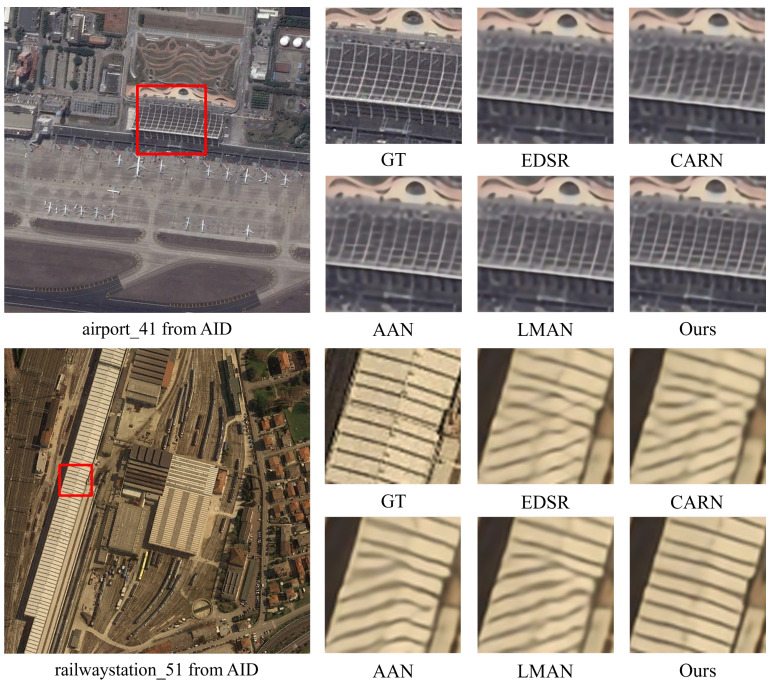
Qualitative comparison of state-of-the-art methods on AID dataset.

One crucial task of remote sensing is SISR, which aims to enhance the resolution of satellite images. Higher-resolution images provide more accurate and detailed information about the Earth’s surface, which is crucial for various applications. Therefore, SISR plays a pivotal role in maximizing the usefulness of remote sensing data. To demonstrate the effectiveness of our proposed method in enhancing remote sensing images obtained from satellite sensors, we present the SISR results of different networks in Figure 8. Our network exhibits clear advantages in recovering remotely sensed images, particularly in capturing texture details, lines, and repetitive structures. In contrast, other contrast algorithms often introduce artifacts and blending issues when dealing with remote sensing images that have complex backgrounds. At the same time, our network effectively mitigates blurring artifacts and reconstructs edge details with higher fidelity.

### 4.3. Ablation Study

In Table 3, we present the results of the ablation study for our method. Below, we discuss the ablation results based on the following aspects:

**The influence of the structure configuration.** The primary objective of this study was to efficiently encode local spatial information, model long-range non-local dependencies, and achieve a global receptive field by leveraging the strengths of Convolutional Networks, which provide spatial inductive biases and local connectivity, and Transformers, which offer input-adaptive weighting and global context interaction. In order to validate the effectiveness of the two core modules, namely MLHA and DGSA, we conducted experiments where one module was removed while the other was retained. The results, as presented in Table 3(a), demonstrate a significant decrease in model performance when either of the modules is removed. These findings indicate that the model benefits from both the global interaction introduced by the DGSA module and the fine-grained local modeling achieved by MLHA.

**The influence of the MLHA part.** In the initial layers of our model, we utilize MLHA to efficiently encode local spatial information. This is achieved by feeding each sub-branch with a specific subset of the complete feature. The effectiveness of the STF strategy is demonstrated in Table 3(b), where it is shown to enhance the explicit learning of distinct feature patterns within the network, leading to improved performance compared to models trained without the STF strategy.

**The influence of the DGSA part.** In the deeper layers of our model, we introduce DGSA to effectively model long-range non-local dependencies and achieve a global receptive field of H × W. To reduce redundancy in attention maps and mitigate interference from cluttered backgrounds, we employ the MTS strategy, which selects multiple top-*k* similar attention matrices and masks out elements with lower weights. In Table 3(c), we display the results of a series of experiments to assess the effectiveness of the DGSA module. These experiments include scenarios with no sparse attention (w/o top-k), sparse attention (w/top-k), and sparse attention with the MTS strategy (top-k with MTS). The results of these experiments indicate that employing sparse attention with the MTS strategy leads to improved performance.

**The influence of the design of LKCS in the MLHA part.** We conducted an experiment to verify the effectiveness of three LKCS modules in our MLHA. Specifically, each LKCS module consists of three convolution layers: DW-Conv layer, DW-D-Conv layer, and Conv layer. The three LKCS modules differ in the kernel size of the three convolution layers they contain. In the first LKCS module, the kernel sizes of the three convolution layers are 3, 5, and 1. In the second LKCS module, The kernel sizes of the three convolution layers are 5, 7, and 1. And in the third LKCS module, the kernel sizes of the three convolution layers are 7, 9, and 1. We wanted to show the effectiveness of extracting features using different kernel sizes. We conducted the experiments, in which the three LKCS modules were exactly the same. The kernel sizes of the three convolution layers in all three LKCS modules were set to 5, 7, and 1. The results are shown in Table 3(d), which shows the effectiveness of our proposed LKCS module.

### 4.4. Application

There are many potential applications of the Lightweight Image Super-Resolution approach. For example, in surveillance, SR techniques can enhance video resolution, making images sharper and clearer so that details, such as facial features and licence plate numbers, can be more easily identified, thus enhancing security. In medical imaging, SR technology can improve the clarity of medical images and help doctors diagnose conditions more accurately. In the field of satellite imagery, SR technology can improve image quality and make remote sensing data analysis more accurate, which is used in environmental monitoring, urban planning, and other fields. The lightweight SR method is particularly suitable for resource-constrained devices and real-time processing scenarios due to its low computation and storage requirements.

## 5. Conclusions

The aim of this study is to develop a lightweight and high-performance network for SISR by effectively combining the strengths of Transformers and Convolutional Networks. To achieve this objective, we propose a novel lightweight SISR method called LGUN. LGUN focuses on encoding local spatial information within MLHA and utilizes the Split–Transform–Fusion (STF) strategy to facilitate the learning of diverse patterns. Additionally, it models global context dependencies through the core module: DGSA. DGSA selects multiple top-*k* similar attention matrices and masks out elements with lower weights, thereby reducing redundancy in attention maps and suppressing interference from cluttered backgrounds. The experimental results, evaluated on popular benchmarks, demonstrate the superior quantitative and qualitative performance of our method.

## Figures and Tables

**Table 1:** Hyper-parameters of the training process.

Training Config	Settings
Random rotation	($90^{\circ }$, $180^{\circ }$, $270^{\circ }$)
Random flipping	Horizontal
Patch size	64 × 64
Batch size	16
Base learning rate	5 × 10^−4^
Optimizer momentum	$\beta_{1}$ = 0.9, $\beta_{2}$ = 0.999
Weight decay	1 × 10^−4^
Learning rate schedule	Cosine decay
Learning rate bound	1 × 10^−7^

**Table 2:** Quantitative comparison with SOTA methods on five popular benchmark datasets. **Thicker** text indicates the best results. ‘Multi-Adds’ is calculated with a 1280 × 720 HR image. The bold font shows the best value in every group.

Method	Scale	#Params (K)	Multi-Adds (G)	Set5	Set14	BSDS100	Urban100	Manga109
Bicubic	×2	∖	∖	33.66/0.9299	30.24/0.8688	29.56/0.8431	26.88/0.8403	30.80/0.9339
SRCNN (TPAMI’14) [15]	×2	57	52.7	36.66/0.9542	32.45/0.9067	31.36/0.8879	29.50/0.8946	35.60/0 9663
VDSR (CVPR’16) [11]	×2	665	612.6	37.53/0.9590	33.05/0.9130	31.90/0.8960	30.77/0.9140	37.22/0.9750
DRCN (CVPR’16) [65]	×2	1774	9788.7	37.63/0.9588	33.04/0.9118	31.85/0.8942	30.75/0.9133	37.55/0.9732
LapSRN (CVPR’17) [66]	×2	813	29.9	37.52/0.9591	33.08/0.9130	31.08/0.8950	30.41/0.9101	37.27/0.9740
MemNet (ICCV’17) [67]	×2	677	623.9	37.78/0.9597	33.28/0.9142	32.08/0.8978	31.31/0.9195	37.72/0.9740
IDN (CVPR’18) [69]	×2	553	127.7	37.83/0.9600	33.30/09148	32.08/0.8985	31.27/0.9196	38.01/0.9749
CARN (ECCV’18) [70]	×2	1592	222.8	37.76/0.9590	33.52/0.9166	32.09/0.8978	31.92/0.9256	38.36/0.9765
EDSR-baseline (CVPR’19) [12]	×2	1370	316	37.99/0.9604	33.57/0.9175	32.16/0.8994	31.98/0.9272	38.54/0.9769
SRFBN-S (CVPR’19) [68]	×2	282	574.4	37.78/0.9597	33.35/0.9156	32.00/0.8970	31.41/0.9207	38.06/0.9757
FALSRA (ICPR’21) [18]	×2	1021	234.7	37.82/0.9595	33.55/0.9168	32.12/0.8987	31.93/0.9256	-
SMSR (CVPR’21) [71]	×2	985	131.6	38.00/0.9601	33.64/09179	32.17/0.8990	32.19/0.9284	38.76/0.9771
A2N (arXiv’19) [72]	×2	1036	247.5	38.06/0.9608	33.75/09194	32.22/09002	32.43/0.9311	38.87/0.9769
LMAN (TBC’21) [26]	×2	1531	347.1	38.08/0.9608	33.80/0.9023	32.22/0.9001	32.42/0.9302	38.92/0.9772
SwinIR (ICCV’21) [32]	×2	878	243.7	38.14/0.9611	33.86/0.9206	32.31/0.9012	**32.76/0.9340**	39.12/0.9783
B-GSCN 10 (KBS’21) [74]	×2	1490	343	38.04/0.9606	33.64/0.9182	32.19/0.8999	32.19/0.9293	38.64/0.9771
DRSDN (KBS’21) [24]	×2	1055	243.1	38.06/0.9607	33.65/0.9189	32.23/0.9003	32.40/0.9308	-
FPNet (TCSVT’22) [75]	×2	1615	-	38.13/0.9619	33.83/0.9198	32.29/0.9018	32.04/0.9278	-
NGswin (CVPR’23) [73]	×2	998	140.4	38.05/0.9610	33.79/0.9199	32.27/0.9008	32.53/0.9324	38.97/0.9777
**LGUN (Ours)**	×2	675	141.1	**38.24/0.9618**	**33.93/0.9208**	**32.34/0.9027**	32.65/0.9322	**39.38/0.9786**
Bicubic	×3	∖	∖	30.39/0.8682	27.55/0.7742	27.21/0.7385	24.46/0.7349	26.95/0.8556
SRCNN (TPAMI’14) [15]	×3	57	52.7	32.75/0.9090	29.30/0.8215	28.41/0.7863	26.24/0.7989	30.48/0.9117
VDSR (CVPR’16) [11]	×3	665	612.6	33.67/0.9210	29.78/0.8320	28.83/0.7990	27.14/0.8290	32.01/0.9340
DRCN (CVPR’16) [65]	×3	1774	9788.7	33.82/0.9226	29.76/0.8311	28.80/0.7963	27.14/0.8279	32.24/0.9343
MemNet (ICCV’17) [67]	×3	677	623.9	34.09/0.9248	30.01/0.8350	28.96/0.8001	27.56/0.8376	32.51/0.9369
IDN (CVPR’18) [69]	×3	553	57	34.11/0.9253	29.99/0.8354	28.95/0.8013	27.42/0.8359	3271/0.9381
CARN (ECCV’18) [70]	×3	1592	118.8	34.29/0.9255	30.29/0.8407	29.06/0.8034	28.06/0.8493	33.50/0.9440
EDSR-baseline (CVPR’19) [12]	×3	1555	160	34.37/0.9270	30.28/0.8417	29.09/0.8052	28.15/0.8527	33.45/0.9439
SRFBN-S (CVPR’19) [68]	×3	375	686.4	34.20/0.9255	30.10/0.8372	28.96/0.8010	27.66/0.8415	33.02/0.9404
SMSR (CVPR’21) [71]	×3	993	67.8	34.40/0.9270	30.33/0.8412	29.10/0.8050	28.25/0.8536	33.68/0.9445
A2N (arXiv’19) [72]	×3	1036	1175	34.47/0.9279	30.44/0.8437	29.14/0.8059	28.41/0.8570	33.78/0.9458
LMAN (TBC’21) [26]	×3	1718	173.8	34.56/0.9286	30.46/0.8439	29.17/0.8067	28.47/0.8576	34.00/0.9470
SwinIR (ICCV’21) [32]	×3	886	109.5	34.60/0.9289	30.54/0.8463	29.20/0.8082	**28.66/0.8624**	33.98/090978
B-GSCN 10 (KBS’21) [74]	×3	1510	154	34.30/0.9271	30.35/0.8425	29.11/0.8035	28.20/0.8535	33.54/0.9445
DRSDN (KBS’21) [24]	×3	1071	109.8	34.48/0.9282	30.41/0.8445	29.17/0.8072	28.45/0.8589	-
FPNet (TCSVT’22) [75]	×3	1615	-	34.48/0.9285	30.53/0.8454	29.20/0.8086	28.19/0.8534	-
NGswin (CVPR’23) [73]	×3	1007	66.6	34.52/0.9282	30.53/0.8456	29.19/0.8078	28.52/0.8603	33.89/0.9470
**LGUN (Ours)**	×3	684	63.5	**34.60/0.9292**	**30.54/0.8458**	**29.25/0.8102**	28.53/0.8586	**34.26/0.9480**
Bicubic	×4	∖	∖	28.42/0.8104	26.00/0.7027	25.96/0.6675	23.14/0.6577	24.89/0.7866
SRCNN(TPAMI’14) [15]	×4	57	52.7	30.48/0.8628	27.50/0.7513	26.90/0.7101	24.52/0.721	27.58/0.85555
VDSR(CVPR’16) [11]	×4	665	612.6	31.35/0.8830	28.02/0.7680	27 29/0.7260	25.18/0.7540	28.83/0.8870
DRCN(CVPR’16) [65]	×4	1774	9788.7	31.53/0.8854	28.02/0.7670	27.23/0.7233	25.18/0.7524	28.93/0.8854
LapSRN(CVPR’17) [66]	×4	813	149.4	31.54/0.8850	28.19/0.7720	27.32/0.7270	25.21/0.7560	29.09/0.8900
MemNet(ICCV’17) [67]	×4	677	623.9	31.74/0.8893	28.26/0.7723	27.40/0.7281	25.50/0.7630	29.42/0.8942
IDN(CVPR’18) [69]	×4	553	32.3	31.82/0.8903	28.25/0.7730	27.41/0.7297	25.41/0.7632	29.41/0.8942
CARN(ECCV’18) [70]	×4	1592	90.9	32.13/0.8937	28.60/0.7806	27.58/0.7349	26.07/0.7837	30.47/0.9084
EDSR-baseline(CVPR’19) [12]	×4	1518	114	32.09/0.8938	28.58/0.7813	27.57/0.7357	26.04/0.7849	30.35/0.9067
SRFBN-S(CVPR’19) [68]	×4	483	852.9	31.98/0.8923	28.45/0.7779	27.44/0.7313	25.71/0.7719	29.91/0.9008
SMSR(CVPR’21) [71]	×4	1006	41.6	32.12/0.8932	28.55/0.7808	27.55/0.7351	26.11/0.7868	30.54/0.9085
A2N(arXiv’19) [72]	×4	1047	72.4	32.30/0.8966	28.71/0.7842	27.61/0.7374	26.27/0.7920	30.67/0.9110
LMAN(TBC’21) [26]	×4	1673	122.0	32.40/0.8974	28.72/0.7842	27.66/0.7388	26.36/0.7934	30.84/0.9129
SwinIR(ICCV’21) [32]	×4	897	61.7	32.44/0.8976	28.77/0.7858	27.69/0.7406	26.47/0.7980	30.92/0.9151
B-GSCN 10(KBS’21) [74]	×4	1530	88	32.18/0.8950	28.60/0.7821	27.59/0.7364	26.12/0.7872	30.50/0.9080
DRSDN(KBS’21) [24]	×4	1095	63.1	32.28/0.8962	28.64/0.7836	27.64/0.7388	26.30/0.7933	-
FPNet(TCSVT’22) [75]	×4	1615	-	32.32/0.8962	28.78/0.7856	27.66/0.7394	26.09/0.7850	-
NGswin(CVPR’23) [73]	×4	1019	36.4	32.33/0.8963	28.78/0.7859	27.66/0.7396	26.45/0.7963	30.80/0.9128
**LGUN (Ours)**	×4	696	36.4	**32.63/0.9008**	**28.94/0.7897**	**27.82/0.7458**	**26.88/0.8084**	**31.52/0.9183**

**Table 3:** Ablation experiments on the micro structure design. The bold font shows the best value in every group.

(a) Results for the MLHA and DGSA modules.					
LGU	Set5	Set14	BSDS100	Urban100	Manga109
	PSNR/SSIM	PSNR/SSIM	PSNR/SSIM	PSNR/SSIM	PSNR/SSIM
w/o MLHA	38.19/0.9616	33.84/0.9199	32.28/0.9018	32.49/0.9307	39.31/0.9784
w/o DGSA	38.15/0.9612	33.65/0.9180	32.25/0.9014	32.18/0.9284	39.11/0.9780
w MLHA + DGSA (Ours)	**38.24/0.9618**	**33.93/0.9208**	**32.34/0.9027**	**32.65/0.9322**	**39.38/0.9786**
(b) Results for the STF strategy in MLHA.					
MLHA	Set5	Set14	BSDS100	Urban100	Manga109
	PSNR/SSIM	PSNR/SSIM	PSNR/SSIM	PSNR/SSIM	PSNR/SSIM
w/o STF	38.20/0.9616	33.89/0.9200	32.30/0.9020	32.48/0.9309	39.28/0.9781
w STF (Ours)	**38.24/0.9618**	**33.93/0.9208**	**32.34/0.9027**	**32.65/0.9322**	**39.38/0.9786**
(c) Results for the MTS strategy in DGSA.					
DGSA	Set5	Set14	BSDS100	Urban100	Manga109
	PSNR/SSIM	PSNR/SSIM	PSNR/SSIM	PSNR/SSIM	PSNR/SSIM
w/o top-*k*	38.21/0.9615	33.87/0.9201	32.32/0.9024	32.56/0.9316	39.30/0.9785
w top-*k*	38.22/0.9616	33.90/0.9203	32.32/0.9024	32.57/0.9317	39.34/0.9786
top-*k* with MTS (Ours)	**38.24/0.9618**	**33.93/0.9208**	**32.34/0.9027**	**32.65/0.9322**	**39.38/0.9786**
(d) Results for the effectiveness of LKCS modules in MLHA.					
MLHA	Set5	Set14	BSDS100	Urban100	Manga109
	PSNR/SSIM	PSNR/SSIM	PSNR/SSIM	PSNR/SSIM	PSNR/SSIM
Identical LKCS	38.11/0.9609	33.62/0.9175	32.19/0.9008	32.13/0.9277	39.05/0.9772
Different LKCS (Ours)	**38.24/0.9618**	**33.93/0.9208**	**32.34/0.9027**	**32.65/0.9322**	**39.38/0.9786**

## Data Availability

The public data used in this work are listed here: Flickr2K [12], Set5 [60], Set14 [61], Urban100 [63], BSDS100 [62], Manga109 [64] and DIV2K [77].
